# A Second Look at the Association between Gender and Mortality on Antiretroviral Therapy

**DOI:** 10.1371/journal.pone.0142101

**Published:** 2015-11-12

**Authors:** Serena P. Koenig, Alexandra Bornstein, Karine Severe, Elizabeth Fox, Jessy G. Dévieux, Patrice Severe, Patrice Joseph, Adias Marcelin, Dgndy Alexandre Bright, Ngoc Pham, Pierre Cremieux, Jean William Pape

**Affiliations:** 1 Haitian Study Group for Kaposi’s Sarcoma and Opportunistic Infections (GHESKIO), Port-au-Prince, Haiti; 2 Division of Global Health Equity, Brigham and Women’s Hospital, Harvard Medical School, Boston, MA, United States of America; 3 Analysis Group, Boston, MA, United States of America; 4 Division of Nutritional Sciences, Cornell University, Ithaca, NY, United States of America; 5 AIDS Prevention Program, Florida International University, Miami, FL, United States of America; 6 Center for Global Health, Weill Cornell Medical College, New York, NY, United States of America; University of Athens, Medical School, GREECE

## Abstract

**Objective:**

We assessed the association between gender and mortality on antiretroviral therapy (ART) using identical models with and without sex-specific categories for weight and hemoglobin.

**Design:**

Cohort study of adult patients on ART.

**Setting:**

GHESKIO Clinic in Port-au-Prince, Haiti.

**Participants:**

4,717 ART-naïve adult patients consecutively enrolled on ART at GHESKIO from 2003 to 2008.

**Main Outcome Measure:**

Mortality on ART; multivariable analyses were conducted with and without sex-specific categories for weight and hemoglobin.

**Results:**

In Haiti, male gender was associated with mortality (OR 1.61; 95% CI: 1.30–2.00) in multivariable analyses with hemoglobin and weight included as control variables, but not when sex-specific interactions with hemoglobin and weight were used.

**Conclusions:**

If sex-specific categories are omitted, multivariable analyses indicate a higher risk of mortality for males vs. females of the same weight and hemoglobin. However, because males have higher normal values for weight and hemoglobin, the males in this comparison would generally have poorer health status than the females. This may explain why gender differences in mortality are sometimes observed after controlling for differences in baseline variables when gender-specific interactions with weight and hemoglobin are omitted.

## Introduction

Nearly 10 million people were receiving antiretroviral therapy (ART) in low and middle-income countries by the end of 2012, an estimated 61% of patients who were eligible under the 2010 World Health Organization (WHO) guidelines [1]. Though ART scale-up is widely viewed as highly successful, questions have been raised about gender equity in access to services [2–5]. Women have been prioritized by funding agencies, international organizations, and local governments, with a focus on the prevention of mother-to-child transmission of HIV [6]. These efforts have resulted in proportionally greater numbers of women receiving HIV testing and ART [7].

Male gender is associated with late HIV testing and more advanced disease at ART initiation [8–18], which is strongly associated with poorer outcomes [19]. Even after controlling for differences in disease severity and other baseline characteristics, multiple studies have found that males have higher mortality after ART initiation [8, 10–13, 20–33]. Proposed reasons for this mortality difference include inequality for males in health care systems in low and middle-income countries, poorer treatment adherence, irregular clinic attendance due to work-related responsibilities, a higher baseline mortality rate for males in the general population, potentially biological differences, and other unmeasured confounders or uncharacterized mechanisms that lead to poorer outcomes for males [2, 3, 5, 8, 12, 34–37].

In Haiti, we found differing results in two studies on predictors of mortality after ART initiation. The earlier study included gender-specific weight quartiles, and did not find an association between male gender and mortality (hemoglobin was not included in the analyses) [38]. The later study included weight and hemoglobin as continuous variables, and found that male gender was associated with higher mortality [39]. We reviewed the literature, and found that many other ART outcomes studies also included weight and hemoglobin without sex-specific categories [8–12, 20–23, 30, 31, 33]. These analyses compare mortality among males vs. females with the same weight and hemoglobin. Due to normal sex differences in these variables, this is not a comparison of males and females of equivalent health status. We therefore repeated the analyses for our later study, adding sex-specific categories for weight and hemoglobin.

## Methods

### Study Setting and Participants

The Haitian Group for the Study of Kaposi’s Sarcoma and Opportunistic Infections is a Haitian non-governmental organization, and the oldest and largest provider of HIV services in the Caribbean. GHESKIO has provided voluntary counseling and testing (VCT) for HIV since 1985. This study included all ART-naïve HIV-infected patients 13 years or older who were consecutively enrolled on ART from February 1, 2003 (when ART became widely available at GHESKIO) to December 31, 2008. Patients were followed until December 31, 2009.

### Data Collection and Statistical Analyses

Data were extracted from the GHESKIO electronic medical record and entered into a Microsoft Access database (Microsoft, Redmond, WA). Baseline weight, hemoglobin, and CD4 cell count were defined as the measurement closest to the date of ART initiation, but not more than six months before or two weeks afterwards. Death was ascertained by chart review, phone calls to next of kin, and home visits. Patients were considered to be alive and in-care if they were not known to be dead and had at least one visit within 6 months of the study endpoint. All analyses were conducted using SAS version 9.3 (SAS Institute, Cary, NC). Patients who were lost to care or transferred to other clinics were censored at their last visit.

Differences between males and females in age, education, income, TB status at ART initiation, initial ART regimen (drug class), and baseline weight, hemoglobin and CD4 cell count were compared using the Chi-square test for binary variables and Wilcoxon rank-sum test for continuous variables. Cox proportional hazards models were used to assess the relationship between baseline demographic and clinical variables and time to death after ART initiation. Gender, income, education, initial ART regimen, and active TB co-infection at ART initiation were defined as binary variables; and hemoglobin, weight, age and CD4 cell count were defined as continuous variables. In the multivariable models we included all variables significant at the 0.20 level in univariable analyses.

We included weight and hemoglobin in the analyses of all-cause mortality using five different models. In Model 1, we included baseline weight and hemoglobin as control variables, without specifying these variables differently for males and females; the interpretation of this model is the risk of mortality for males vs. females who presented with the same baseline weight and hemoglobin. In Model 2, we included weight and hemoglobin using differences from the sex-specific median; the interpretation of this model is the risk of mortality for males vs. females who have the same deviation from the sex-specific cohort medians for these variables. The goal of Model 3 was to compare mortality for males vs. females with the same deviation from a healthy sex-specific standard for weight and hemoglobin. Since there is no standard measure of healthy weight without the inclusion of height, and height was not measured, we used deviation from gender-specific median for weight. For hemoglobin, we used the WHO definition of anemia as a binary variable (cut-off of 12 g/dL for women and 13 g/dL for men) [40]. In Model 4, we used sex-specific quartiles for weight and hemoglobin; the interpretation of this model is the risk of mortality for males vs. females who fall within the same sex-specific cohort quartiles for these variables. In Model 5, we excluded any weight or hemoglobin-related variables but included all other variables as in the other models. Our rationale for the inclusion of Model 5 is for comparability with other studies that did not include these variables. We included an anonymized dataset (see S1 Appendix) that includes all data needed to reproduce these results.

### Ethics Statement

This study was approved by the ethics committees of GHESKIO, Weill Cornell Medical College, and Brigham and Women’s Hospital. It was not feasible to obtain informed consent for this retrospective study, but patient information was anonymized and de-identified prior to analysis.

## Results

The 4,717 ART-naïve HIV-infected patients age 13 years or older who were consecutively enrolled on ART during the study period were included. Characteristics for males and females are summarized in Table 1. The cohort included 2,151 males (46%) and 2,566 females (54%). Males were older (median age 40 vs. 36 years; p<0.0001), less likely to live on ≤US$365/year (53% vs. 71%; p<0.0001), less likely to have no school or primary school only (43% vs. 56%; p<0.0001), and more likely to have TB at ART initiation (6% vs. 4%; p<0.0001). At baseline, males had higher median body weight (57 vs. 50 kilograms; p<0.0001), higher median hemoglobin (11.0 vs. 10.0 g/dl; p<0.0001), and lower median CD4 counts (134 vs. 105 cells/mm^3^). The median follow-up time was 828 days (interquartile range (IQR): 388 to 1,505 days) for males and 825 days (IQR: 395 to 1,492 days) for females. Of the 4717 patients in the cohort, 293 males (14%) and 305 females (12%) died during the study period; 602 patients (13%) were lost to follow-up, including 288 males (13%) and 314 females (12%). The LTFU rates were not statistically significantly different between sexes (p = 0.2375).

**Table 1:** Baseline Patient Characteristics by Gender.

Variable*	Male (n = 2,151)	Female (n = 2,566)	P-value
Median Age (IQR)**	40 (33 to 46)	36 (33 to 43)	<0.0001
No school or primary school only—no. (%)	931 (43)	1,435 (56)	<0.0001
Income ≤US$365/year—no. (%)	1,148 (53)	1,813 (71)	<0.0001
Median weight—kilograms (IQR)**	57 (50–64)	50 (44–58)	<0.0001
Median hemoglobin—g/dl (IQR)**	11.0 (9.6–12.2)	10.0 (8.9–11.0)	<0.0001
Hemoglobin <8.0 (g/dl)–no. (%)	116 (5)	234 (9)	<0.0001
Hemoglobin 8.0–10.9 (g/dl)–no. (%)	835 (39)	1,422 (55)	
Hemoglobin 11.0–12.9 (g/dl)–no. (%)	694 (32)	585 (23)	
Hemoglobin ≥13.0	287 (13)	46 (2)	
Median CD4 cell count (IQR)**	105 (39–182)	134 (59–198)	<0.0001
CD4 cell count <50 cells/mm^3^	561 (29)	513 (22)	<0.0001
CD4 cell count 50–99 cells/mm^3^	372 (19)	404 (17)	
CD4 cell count 100–199 cells/mm^3^	648 (33)	853 (36)	
CD4 cell count ≥ 200 cells/mm^3^	370 (19)	573 (24)	
TB at ART initiation—no. (%)	126 (6)	107 (4)	0.0077
ART regimen includes non-nucleoside reverse transcriptase inhibitor	2,044 (95)	2,431 (95)	0.6567

* Two variables had ≥5% of data missing. There were 219 (10%) missing hemoglobin results for males and 279 (11%) for females. There were 200 (9%) missing CD4 count results for males and 223 (9%) for females.

**Median values and percentages are computed using the number of patients with non-missing values.

### Univariable and Multivariable Analyses without Sex-Specific Categories for Weight and Hemoglobin

We conducted Cox proportional hazards regression with baseline weight and hemoglobin as non-sex specific variables. In the univariable analysis, gender, age, income, weight, hemoglobin, CD4 cell count, and TB status at ART initiation had p-values <0.20, and were included in the multivariable analyses. Education and ART regimen were not associated with mortality (p = 0.503 and 0.674, respectively) and were excluded from further analyses.

The sample size for the multivariable analysis was 3761 patients. Nine hundred fifty-six patients were missing at least one of the independent variables. Male gender (adjusted hazard ratio [aHR] 1.61; 95% CI: 1.30–2.00) and older age were associated with mortality (aHR 1.22 for every 10-year increase in age; 95% CI: 1.11–1.34). Higher baseline weight (aHR 0.63 for every 10-kilogram increase in weight; 95% CI: 0.56–0.70), hemoglobin (aHR 0.87; 95% CI: 0.82–0.92), and CD4 cell count (aHR 0.87 for every 50 CD4 cells; 95% CI: 0.82–0.93) were associated with improved survival (see Table 2, Model 1).

**Table 2:** Cox Proportional Hazards Models of Mortality on ART using Different Methods of Categorizing Weight and Hemoglobin (Models 1, 2, and 3).

Variable	Model 1: Weight and hemoglobin as continuous variables		Model 2: Deviation from gender-specific median for weight and hemoglobin		Model 3: Deviation from gender-specific median weight and gender-specific WHO anemia definition*	
	Adjusted HR (95% CI)	p-value	Adjusted HR (95% CI)	p-value	Adjusted HR (95% CI)	p-value
Male gender	1.61 (1.30–2.00)	<0.0001	1.05 (0.85–1.29)	0.6437	1.04 (0.84–1.27)	0.7444
Age (unit = 10 years)	1.22 (1.11–1.34)	<0.0001	1.22 (1.11–1.34)	<0.0001	1.22 (1.11–1.34)	<0.0001
Income ≤$US365 per year	1.22 (0.97–1.54)	0.0884	1.22 (0.97–1.54)	0.0884	1.22 (0.97–1.54)	0.0884
Weight (unit = 10 kilograms)	0.63 (0.56–0.70)	<0.0001	0.63 (0.56–0.70)	<0.0001	0.63 (0.56–0.70)	<0.0001
Hemoglobin (g/dl)	0.87 (0.82–0.92)	<0.0001	0.87 (0.82–0.92)	<0.0001	0.87 (0.82–0.92)	<0.0001
CD4 cell count (unit = 50 cells)	0.87 (0.82–0.93	<0.0001	0.87 (0.82–0.93)	<0.0001	0.87 (0.82–0.93)	<0.0001
TB at ART initiation	1.39 (0.97–1.98)	0.0711	1.39 (0.97–1.99)	0.0711	1.39 (0.97–1.99)	0.0711

*Difference from gender-specific median for weight, and World Health Organization definition of anemia (≤12.0 g/dl for women and ≤13.0 g/dl for men)

### Multivariable Analyses Using Sex-Specific Categories for Weight and Hemoglobin

We conducted three different additional analyses using sex-specific categories for weight and hemoglobin, as described above (Models 2, 3, and 4). As illustrated in Tables 2 and 3, in each of these three models, older age was associated with mortality. Higher baseline weight, hemoglobin, and CD4 cell count were associated with improved survival. Male gender was not a predictor of mortality.

**Table 3:** Cox Proportional Hazards Model of Mortality on ART Using Gender-Specific Quartiles for Hemoglobin and Weight (Model 4).

Variable	Model 4: Adjusted HR (95% CI)	p-value
Male gender	1.12 (0.91–1.38)	0.2813
Age (unit = 10 years)	1.18 (1.08–1.30)	<0.0001
Income ≤$US365/year	1.30 (1.03–1.63)	0.0269
Weight—reference group bottom quartile		
— Quartile 2 for gender	0.49 (0.37–0.64)	<0.0001
— Quartile 3 for gender	0.41 (0.31–0.54)	<0.0001
— Quartile 4 for gender	0.39 (0.28–0.54)	<0.0001
Hemoglobin—reference group bottom quartile		
— Quartile 2 for gender	0.58 (0.44–0.75)	<0.0001
— Quartile 3 for gender	0.66 (0.50–0.87)	0.0029
— Quartile 4 for gender	0.47 (0.34–0.66)	<0.0001
CD4 cell count (unit = 50 cells)	0.87 (0.82–0.92)	<0.0001
TB at ART initiation	1.36 (0.95–1.95)	0.0910

We repeated the analyses with the exclusion of any baseline hemoglobin or weight control variables, for comparison with studies that did not include these variables in the analyses (see Table 4, Model 5). With the exclusion of these variables, male gender was not associated with higher mortality. Older age and income ≤$US 365 per year were associated with higher mortality, and higher CD4 cell count was associated with improved survival.

**Table 4:** Cox Proportional Hazards Models of Mortality on ART with the Exclusion of Weight and Hemoglobin (Model 5).

Variable	Model 5: No interaction	
	Adjusted HR (95% CI)	p-value
Male gender	1.15 (0.93–1.41)	0.2003
Age (unit = 10 years)	1.16 (1.05–1.28)	0.0037
Income ≤$US365 per year	1.53 (1.22–1.92)	0.0002
CD4 cell count (unit = 50 cells)	0.83 (0.78–0.88)	<0.0001
TB at ART initiation	1.61 (1.13–3.00)	0.0886

## Discussion

In Haiti, as in most other resource-poor settings, male gender is associated with advanced disease at ART initiation [8–18]. However, we found that after controlling for baseline differences, the association between male gender and mortality on ART depended on whether or not sex-specific categories were used for weight and hemoglobin. Without the use of sex-specific categories, we found that the adjusted hazard ratio of mortality for males was 1.61 (1.30–2.00). Male gender was not associated with mortality if sex-specific categories were used for weight and hemoglobin, or these variables were excluded from the analysis.

Many studies from resource-poor settings have described higher rates of mortality for males on ART [8–13, 17, 20–33, 41, 42]. However, these studies did not use sex-specific categories for both weight and hemoglobin, and therefore indicate a higher mortality for males vs. females of the same weight and hemoglobin. As the males in this comparison would generally have poorer health status at equal baseline weight and hemoglobin levels, higher mortality would be expected.

Six of these studies from China, South Africa, and Tanzania included body weight in the analyses without sex-specific categories [8, 10, 11, 22, 29, 33]. Though BMI is a superior predictor of nutritional status as it includes height, it is not always available, and weight is used as a proxy. All six of these studies found that male gender was associated with higher mortality. Studies from Cambodia, Thailand, Burkina Faso, Cameroon, Cote d’Ivoire, Malawi, Nigeria and Tanzania used body mass index, and none of these used sex-specific categories [9, 12, 20, 21, 23, 27, 28, 30, 31]. BMI is commonly used as a predictor of disease in high BMI ranges without sex-specific categories. However, there is limited evidence linking low BMI ranges to disease outcomes in the developing country context, particularly when sex-specific categories are not used [43–45]. In addition, BMI poorly discriminates between body composition phenotypes, i.e. bone mass, fat mass and lean muscle mass [43, 46], and significant sex differences exist in body composition phenotypes [43, 47, 48]. Thus, the same BMI value may have different implications for the health and nutritional status of HIV-infected men and women.

Most studies from resource-poor settings that included hemoglobin did not include sex-specific categories [9, 11, 12, 20–23, 30, 31]. However, criteria for anemia are sex-specific. WHO defines anemia as a hemoglobin level below 12 g/dL in females and below 13 g/dL in males [40], and multiple studies evaluating outcomes in other diseases have used sex-specific categories for hemoglobin [49–53]. Most studies that did not use sex-specific hemoglobin categories found an association between male gender and mortality [11, 12, 20–23, 30, 31]. One study from Cambodia did not use sex-specific hemoglobin categories and found no association between gender and mortality, but it included patients who were severely immunocompromised (median CD4 cell count of 20 cells/mm^3^), making comparisons with other cohorts difficult [9]. One South African study included sex-specific categories for hemoglobin, and found an association between male gender and mortality [8]. However, this study included weight as a continuous variable, without sex-specific categories. The authors also compared the gender differential in mortality among patients on ART to the background gender differential in mortality in the South African population. They found that the gender mortality ratio among patients on ART appeared to be smaller than the age-standardized HIV-negative mortality ratio for men vs. women in South Africa, suggesting that higher background mortality rates among males contributed to the association with mortality on ART. Therefore, the finding of the association between male gender and mortality on ART could be misinterpreted.

Several studies from India, Ethiopia, Lesotho, Malawi, South Africa, and Uganda did not include either weight or hemoglobin in the analyses [13, 17, 24–26, 32, 41, 42]. These studies generally involved retrospective cohorts, and data for these variables had not been collected. Hemoglobin and weight provide two additional metrics to proxy the underlying baseline health status of patients, when they are available. Models that exclude hemoglobin and weight should result in a weaker relationship between gender and mortality than models that include these variables without attention to sex-specific differences in healthy values. However, because these studies do not explicitly control for sex-specific differences in healthy weight and hemoglobin levels, they are still likely to overstate male mortality. Some studies that had not included these variables found an association between male gender and mortality on ART [13, 24–26, 32] while others did not [17, 41, 42].

Though our study found that male gender was not associated with higher mortality after controlling for baseline characteristics that proxy for disease severity with attention to sex-specific differences in healthy weight and hemoglobin, this does not diminish concern about equity in access to care for males [2–5, 13, 27, 28]. In our analysis as in others, males present later for HIV testing, and start ART with more advanced disease; such delays in ART initiation are associated with higher morbidity and poorer long-term survival [19]. Innovative outreach efforts for earlier HIV testing and timely ART initiation for males remain critical to maximize HIV treatment outcomes, particularly since our analysis shows that, controlling for baseline characteristics that proxy for disease severity, there is no evidence of lower survival for males relative to females after ART initiation.

Our study was limited by the use of retrospective data from a single country, and by our lack of BMI data. It was also limited by the lack of information about mortality in patients who were LTFU. We did not correct mortality for LTFU in our analyses. Our rate of LTFU (13% with median follow-up time of 27 months) is lower than in many studies from resource-poor settings [54, 55], and was similar in males and females. We wanted to compare our findings to other published studies evaluating the sex differences in ART outcomes. These studies either did not correct mortality for LTFU [10, 11, 20, 22, 27, 28, 30], conducted separate analyses to identify predictors of mortality and LTFU [12, 17, 23–25, 31, 32, 42], or used weighted data or competing risk models (either in the base case analysis or in sensitivity analyses) to correct mortality estimates for those who were LTFU but had died [8, 9, 13, 21, 26, 29, 33, 41].

In comparing our study with others from resource-poor settings that evaluated the association between gender and mortality on ART, it is important to also consider that normal weight and hemoglobin values vary by age and ethnicity. All patients in our cohort were Haitian, of African descent. The median age in our study was 38 years, and we included patients of at least 13 years of age. The median age in other studies ranged from 32 to 39 years [8–13, 17, 20–33, 41, 42], and many included adolescents as well as adult patients [8, 12, 13, 20, 21, 24–28, 32, 33].

The association between gender and ART outcomes is complex, and the reasons for gender differences vary by settings. However, our finding that the association between gender and mortality is no longer observed once sex-specific baseline characteristics are included has relevance for other countries and other diseases.

In summary, we found that males do not have higher mortality after ART initiation, as long as sex-specific categories for weight and hemoglobin are included to allow a comparison of gender-specific survival given similar morbidity levels at baseline. If sex-specific categories are not used, then the hazard ratio must be interpreted as a comparison of males and females with the same weight and hemoglobin but not the same baseline morbidity levels.

## Supporting Information

S1 Appendix Anonymized Dataset. (XLS)
